# CRISPR/Cas inactivation of *RECQ4* increases homeologous crossovers in an interspecific tomato hybrid

**DOI:** 10.1111/pbi.13248

**Published:** 2019-09-30

**Authors:** Ruud A. de Maagd, Annelies Loonen, Jihed Chouaref, Alexandre Pelé, Fien Meijer‐Dekens, Paul Fransz, Yuling Bai

**Affiliations:** ^1^ Bioscience Cluster Plant Developmental Systems Wageningen University & Research Wageningen The Netherlands; ^2^ Bioinformatics Group Wageningen University & Research Wageningen The Netherlands; ^3^ Plant Breeding Wageningen University & Research Wageningen The Netherlands; ^4^ Department of Plant Development and (Epi)Genetics Swammerdam Institute for Life Sciences University of Amsterdam Amsterdam The Netherlands

**Keywords:** meiosis, interspecific crosses, RECQ4, class II crossover pathway, introgression breeding

## Abstract

Crossover formation during meiosis in plants is required for proper chromosome segregation and is essential for crop breeding as it allows an (optimal) combination of traits by mixing parental alleles on each chromosome. Crossover formation commences with the production of a large number of DNA double‐strand breaks, of which only a few result in crossovers. A small number of genes, which drive the resolution of DNA crossover intermediate structures towards non‐crossovers, have been identified in *Arabidopisis thaliana*. In order to explore the potential of modification of these genes in interspecific hybrids between crops and their wild relatives towards increased production of crossovers, we have used CRISPR/Cas9‐mutagenesis in an interspecific tomato hybrid to knockout *RecQ4*. A biallelic *recq4* mutant was obtained in the F1 hybrid of *Solanum lycopersicum* and *S. pimpinellifolium*. Compared with the wild‐type F1 hybrid, the F1 *recq4* mutant was shown to have a significant increase in crossovers: a 1.53‐fold increase when directly observing ring bivalents in male meiocytes microscopically and a 1.8‐fold extension of the genetic map when measured by analysing SNP markers in the progeny (F2) plants. This is one of the first demonstrations of increasing crossover frequency in interspecific hybrids by manipulating genes in crossover intermediate resolution pathways and the first to do so by directed mutagenesis.

**Significance statement:**

Increasing crossover frequency during meiosis can speed up or simplify crop breeding that relies on meiotic crossovers to introduce favourable alleles controlling important traits from wild relatives into crops. Here we show for the first time that knocking out an inhibitor of crossovers in an interspecific hybrid between tomato and its relative wild species using CRISPR/Cas9‐mutagenesis results in increased recombination between the two genomes.

## Introduction

Meiotic recombination is a crucial event in sexual reproduction. In the first place, the formation of crossovers is essential for proper chromosome segregation during gamete formation. Secondly, the reciprocal exchange between homologous chromosomes enables the reshuffling of parental genetic information and transfer of the recombined material to the next generation. This makes meiotic crossover formation an important target in crop breeding. Meiosis starts with the formation of a large number of DNA double‐strand breaks (DSB), most of which are repaired in non‐crossover events and do not result in recombinant chromosomes. In most organisms, and especially in plants, only a few (one to three) DSBs per chromosome are processed into actual crossovers (Lambing *et al*., 2017; Mercier *et al*., 2015). The repair of DSB occurs when one of the ssDNA strands invades into either its sister chromatid or the homologous chromosome facilitating homology‐directed repair. DSB DNA repair via homologous chromosomes occurs through joint molecules that are processed into crossovers or, more frequently into non‐crossovers. Two pathways of crossover formation exist in most organisms: the interfering pathway and the non‐interfering pathway, giving rise to class I and class II crossovers, respectively. The number of class I crossovers is limited by interference, the phenomenon that prevents the occurrence of two crossovers in close proximity (Mercier *et al*., 2005).

Nevertheless, class I crossovers form the majority of crossovers. In the interfering pathway, the ZMM proteins (Zip1‐4, Msh4/5, and Mer3) together with MLH1 (MutL Homolog 1) and HEI10 (Homolog of ENHANCER OF CELL INVASION NO.10) control the formation of class I crossovers. The non‐interfering class II crossovers are generated via an alternative pathway involving the MUS81 (MMS AND UV SENSITIVE 81) nuclease (Osman *et al*., 2003). In *Arabidopsis*, crossover formation in this pathway is strongly limited due to the action of anti‐crossover factors, such as the topoisomerase TOP3α (TOPOISOMERASE 3α), and the DNA helicases RECQ4 (RecQ helicase 4) and FANCM (Fanconi Anaemia of Complementation group M). They may dissolve D‐loops or double DNA Holliday Junctions into non‐crossovers (Mercier *et al*., 2015; Séguéla‐Arnaud *et al*., 2015).

The RECQ family of helicases is an evolutionarily conserved family of proteins, from bacteria to plants and animals. They are ATP‐ and DNA‐dependent helicases, which separate double‐stranded DNA in a 3′ to 5′ direction (Kaiser *et al*., 2017). They are involved in a diversity of functions such as telomere stability, DNA replication, DNA recombination and DNA repair. Their functions are best known in humans, as mutations in *RECQ* genes can lead to developmental abnormalities and diseases (Fu *et al*., 2017). For example, Bloom syndrome, characterised by genome instability, is caused by mutations in human *RecQ4* (*BLM*), and orthologs with similar functions can be readily identified in yeast (*Sgs1*, Slow growth suppressor 1) and plants (Hartung and Puchta, 2006). Brassicaceae, including *Arabidopsis*, contains two orthologs, *RecQ4A* and *RecQ4B* due to recent gene duplication, as do lettuce and sunflower, members of the Asteraceae (Mieulet *et al*., 2018). Both helicases, *RecQ4A* and *RecQ4B* are functional in *Arabidopsis*. *Arabidopsis recq4a* plants, with an intact copy of *RecQ4B*, display sensitivity to mutagens and increased recombination. *RecQ4A*, but not any of the other *Arabidopsis RecQ* genes, including *RecQ4B*, can complement the phenotype of *sgs1* mutants in yeast (Bagherieh‐Najjar *et al*., 2005). This suggests that the two *Arabidopsis* orthologs have diverged functions, raising questions about the function of the ancestral *RecQ4* gene and the single orthologs in other higher plants. Deletion of the single *RecQ4* ortholog in the moss *Physcomitrella patens*, also results in sensitivity to mutagens and somatic hyperrecombination, suggesting that this function was maintained in all plants with a single ortholog, and in *RecQ4A* of *Arabidopsis* (Wiedemann *et al*., 2018). *Recq4ab* double mutants, but not the single mutants, show a sixfold increase in crossover frequency in intraspecific *Arabidopsis* crosses (Séguéla‐Arnaud *et al*., 2015).

The identification of the different components in the crossover pathways enables the manipulation of the frequency and/or position of crossovers. Blocking anti‐crossover factors increases type II crossovers. For example, blocking FANCM function in *Arabidopsis* led to a threefold increase in crossovers (Crismani *et al*., 2012), as does mutation of its co‐factors FANC*M*‐interacting *H*istone *F*old proteins (MHF) 1 and 2 (Girard *et al*., 2014). In parallel to FANCM, TOP3α and the RECQ4A/B helicases function in a tripartite RTR complex (RECQ4/TOP3α/RMI) with RMI (RecQ4‐mediated instability 1), also leading to non‐crossovers. Mutant alleles of *TOP3*α are early lethal or sterile (Hartung *et al*., 2008). However, a specific non‐sense mutation, *top3*α*‐R640X* with intact topoisomerase‐primase and topoisomerase domains, was found in a suppressor screen of an infertile *zmm* mutant. This suppressor mutant was fertile while the mutation in an otherwise wild‐type background led to a 1.5‐fold increase in crossovers in *Arabidopsis* (Séguéla‐Arnaud *et al*., 2015). In a similar screen, *rmi1* mutants were found to restore crossover in *zmm* mutants and increasing crossovers fourfold in a single mutant, establishing *RMI1*'s anti‐crossover action (Séguéla‐Arnaud *et al*., 2017).

Another anti‐crossover factor is the AAA‐ATPase‐encoding gene *FIDGETIN‐Like‐1* (*FIGL1*). Plants with a *figl1‐1* mutation show 1.7‐fold increased crossovers, while in *a figl1‐1 fancm‐1* double mutant even a sixfold increase has been observed (Fernandes *et al*., 2018; Girard *et al*., 2015). In addition, increased doses of the E3 ubiquitin ligase *HEI10* resulted in higher crossover frequencies, which was further increased when combined with *recq4ab* mutations (Serra *et al*., 2018). These results indicate that there is considerable room for improving crossover frequency in plants. In comparison with *Arabidopsis*, crop species have been largely disregarded. So far, knockout of *FANCM* or *RECQ4* orthologs have been tested in the rapeseed, rice, pea and tomato with contrasting results (Blary *et al*., 2018; Mieulet *et al*., 2018). For instance, a mutation of *FANCM* in *Brassica napus* leads to a 1.3‐fold increase versus threefold in *Arabidopsis* and *B. rapa* (Blary *et al*., 2018), although the *B. napus* mutation is not necessarily null and showed a limited effect in intraspecific hybrids compared to pure lines (Fernandes *et al*., 2018). On the other hand, the single *recq4* mutation increases crossovers approximately threefold in all crops tested (Mieulet *et al*., 2018), suggesting that manipulating *RECQ4* may be a universal tool for increasing recombination in plants.

For interspecific crosses, the elevation of crossover frequency can greatly enhance introgression breeding. In tomato (*Solanum lycopersicum*), which can interbreed with a number of wild relative species such as (but not limited to) *S. pimpinellifolium, S. chilense, S. cheesmanii, S. chmielewskii, S. pennellii* and *S. habrochaites*, introgression of pathogen or arthropod resistance genes, as well as of abiotic stress tolerance and fruit quality traits is an important aspect of crop breeding (Rick, 1988; Rick and Chetelat, 1995). Such introgression breeding is often hampered by a lack of sufficient crossovers nearby the desired trait‐encoding gene, leading to co‐introgression of undesirable genes from the wild parent, so‐called linkage drag (Bai and Lindhout, 2007). Thus, there is a large demand for increasing crossover frequencies in interspecific crosses in crops in general, including in tomato breeding.

Here we studied the effect of *RECQ4* on crossover frequency in the interspecific F1 hybrid of *S. lycopersicum* (cv. Moneymaker) x *S. pimpinellifolium*. The tomato genome contains only one ortholog of *RECQ4*. We applied the CRISPR/Cas9 technique to generate mutations in *RECQ4* alleles of both parents directly in the F1 hybrid. Diakinesis analysis of meiocytes in the mutant plant showed a significant increase of chiasmata, which is suggestive of an increase in crossovers during male meiosis. Moreover, the hybrid background allowed the tracing of genome‐wide crossover events by genotyping F2 progeny and thus directly establishing the positive effect of *RECQ4* knockout on homeologous crossovers in a crop breeding intermediate.

## Results

### Identification of a *RECQ4* ortholog in tomato

**Figure 1:**
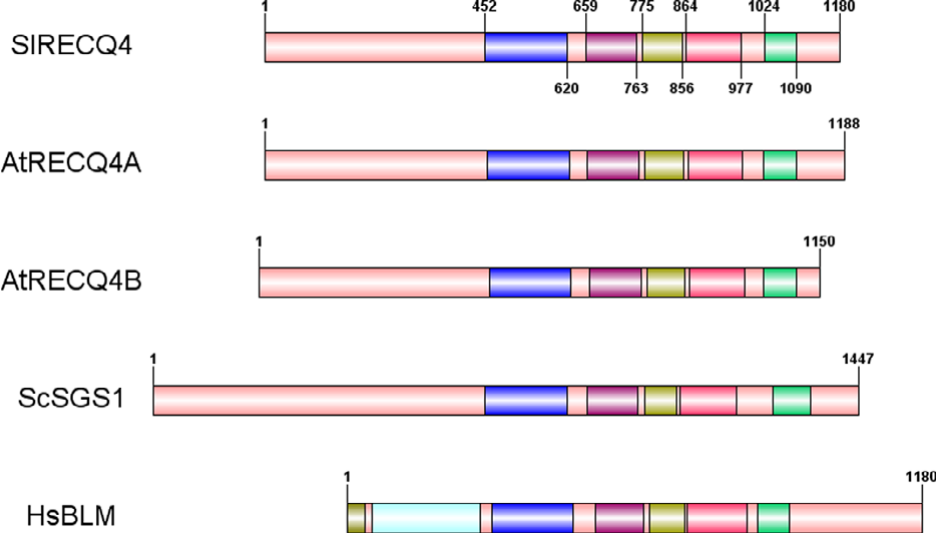
Domain composition of the tomato RECQ4 protein, its *Arabidopsis* orthologs RECQ4A and RECQ4B, *Saccharomyces cerevisiae* SGS1 and human BLM protein. Domains are, in BLM only: Grey: BDHCT; Light Blue: BDHCT‐associated; Common: Dark blue: DEAD‐Box helicase; Purple: Helicase conserved C‐terminal; Yellow: RecQ zinc‐binding; Red: RQC; Green: HRDC.

In *Arabidopsis*,* RECQ4* consists of two paralogs, *RECQ4A* (At1g10930) and *RECQ4B* (At1g60930). Sequences of the closest homologs from *Arabidopsis* and tomato were collected from BlastP searches in Genbank proteins. Grape (*Vitis vinifera*) as a distant relative of tomato and *Arabidopsis* was added to support the analysis. These were used to make a phylogenetic tree from *Arabidopsis* RECQ1‐5, SIM and RECQ‐Like proteins and their putative orthologs in the other two species (Figure S1). A fork with 95% bootstrap value separates *Arabidopsis* RECQ2 and its tomato ortholog from *Arabidopsis* RECQ4A&B and their single tomato ortholog XP_004231337.1 (Genbank mRNA model XM_004231289, 4157 nucleotides; Solanaceae Genomics Network: Solyc01g103960), confirming the analysis of plant RECQ4 proteins published elsewhere (Mieulet *et al*., 2018). These phylogenetic relationships were further confirmed by ortholog searches in Plaza 4.0 (Van Bel *et al*., 2018). The tomato *RECQ4* ortholog, according to its predicted sequence, contains 25 exons encoding a protein of 1180 amino acids. This protein contains, starting from amino acid 451, five conserved protein domains as found in the Pfam database (https://pfam.xfam.org): a DEAD/DEATH box helicase domain (PF00270), a Helicase conserved C‐terminal domain (PF00271), a RecQ zinc‐binding domain (PF16124), the RECQ family‐specific RQC domain (PF09382) and a HRDC (helicase and RNaseD C‐terminal; PF00570) domain. The tomato *RECQ4* has the same domains, and in the same order, in common with the *Saccharomyces cerevisiae* Sgs1 protein and human BLM protein (Figure 1). As found previously (Hartung and Puchta, 2006), only the RECQ2 and RECQ4 orthologs contain the latter two domains. A Blast search of the *S. pimpinellifolium* LA1589 genome (https://Solgenomics.net) revealed the presence of a single *RECQ4* ortholog in this Solanum species with 99.8% nucleotide identity, encoding a protein of identical size with only two amino acids substitutions (T339I, P976T) relative to the tomato RECQ4.

### CRISPR/Cas9‐mutagenesis and selection of a biallelic *recq4* null mutation in an interspecific hybrid

**Figure 2:**
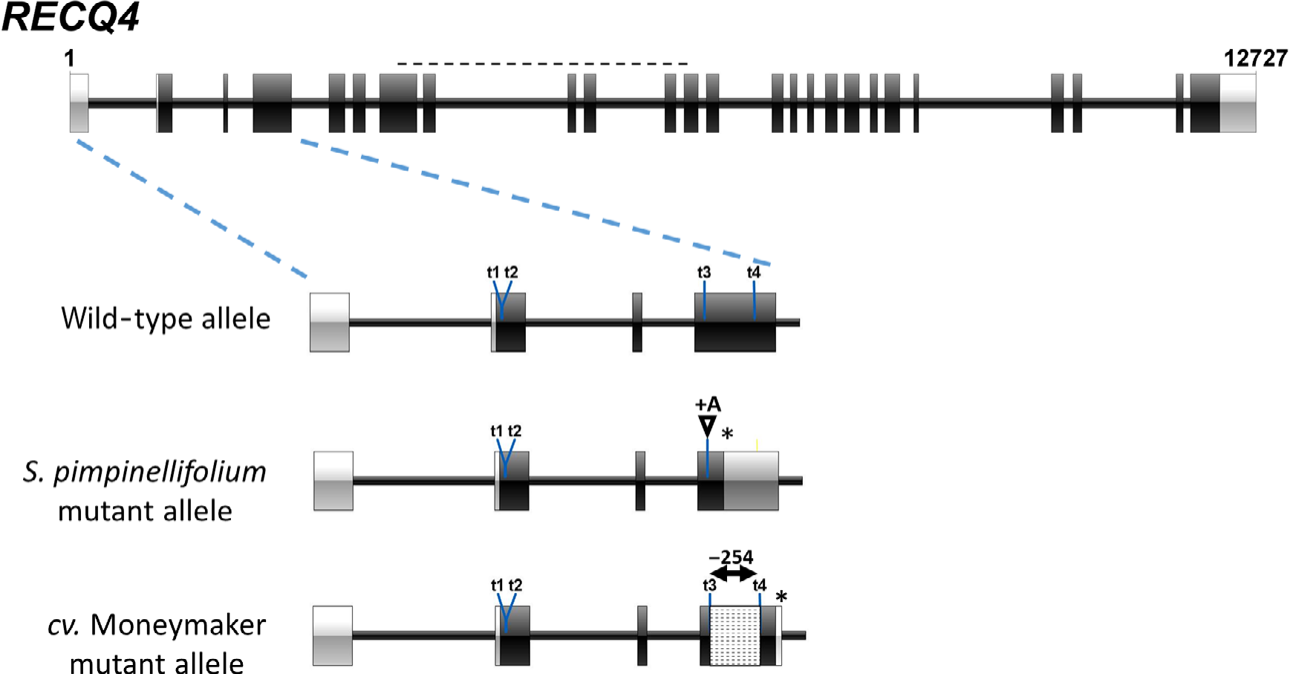
Overview of CRISPR/Cas9 mutagenesis of the tomato *RECQ4* gene in the *Solanum lycopersicum* and *S. pimpinellifolium* parental alleles of the F1 hybrid of the two species. The black horizontal dashed line indicates the extent of the DEAD‐box encoding sequence. t1 to t4 are sgRNA target locations. *Showing the position of the premature stop codon caused by the 1nt‐insertion in the *Solanum pimpinellifolium* mutant allele, and by the 254 bp deletion of the cv. Moneymaker mutant allele, respectively.

In order to examine the effect of RECQ4 on crossover frequency in an interspecific hybrid, we introduced two mutant *RECQ4* alleles in the F1 hybrid *S. lycopersicum* (cv. Moneymaker) X *S. pimpinellifolium* (G1.1554). We generated these mutations using CRISPR/Cas9 and designed four single‐guide RNA's for inclusion in a binary *Cas9*/guideRNA‐expression vector (Brooks *et al*., 2014), which was subsequently used for transformation of the F1 hybrid. The four synthetic‐guide RNA's were targeted to the 5′ end of the *RECQ4* open reading frame, two in exon 1 and two in exon 3 each (Figure 2). The regenerating plants were screened initially by PCR amplification of the targeted region followed by gel electrophoresis, for the occurrence of at least one visible deletion allele. Subsequently, the PCR fragment of the allele without an obvious deletion was Sanger sequenced to screen for smaller INDELS. In this way, we identified a transformant (AL809) with two mutant alleles: a 254 nt deletion between Cas9 targets 3 and 4 in the *S. lycopersicum* allele and a single A insertion at target 4 of the *S. pimpinellifolium* allele (Figure 2). Both mutations result in in‐frame premature stop codons in the third exon and thus express, if any, truncated proteins lacking all conserved domains listed above, which makes it unlikely to exert residual activity. A cDNA fragment encompassing the targeted region of *RECQ4* was amplified from wild‐type (wt) and mutant F1 plant flowers and analysed by gel electrophoresis. Whereas in wt F1 flowers a single band of the expected size was observed, mutant flowers contained a new band representing the product of the deletion allele of cv. Moneymaker (Figure S2a). Sequencing of the upper band revealed that this consisted entirely of the single nucleotide insertion allele of *S. pimpinellifolium* (Figure S2b). Thus, we concluded that the selected mutant no longer produces mRNA's encoding intact RECQ4 protein, although residual activity could not be completely excluded.

### *RECQ4* knockout results in increased frequency of ring bivalents in male meiocytes

**Figure 3:**
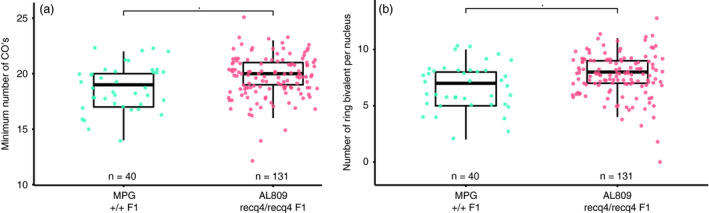
Crossover analysis in male meiocytes of wild type (MPG) and recq4 (AL809) (a) Number of ring bivalents per nucleus and (b) Minimum number crossovers, counted as 12+ number of ring bivalents. Numbers of nuclei counted are 40 for MPG and 131 for AL809, respectively. Asterisks indicate *P*‐value < 0.05 (*t*‐test).

From the mutant F1 plant (AL809, named the *recq4* mutant) four cuttings were taken and grown into flowering plants for microscopic analysis of meiotic aberrations and gametophytic crossover frequency. Crossover frequency was estimated by analysing chiasmata in diakinesis cells and counting ring bivalents, the hallmark of two crossovers occurring at opposing ends of a chromosome pair. Quantification of the ring bivalents revealed significantly more bivalents (7.7 vs 6.7, *P* = 0.008) per nucleus in the *recq4* mutant (Figure 3a) and thus suggesting a higher frequency of crossovers (Figure 3b). The results indicate that *RECQ4* mutation by CRISPR/Cas9 increases the recombination frequency in an interspecific hybrid between tomato and *S. pimpinellifolium*.

**Figure 4:**
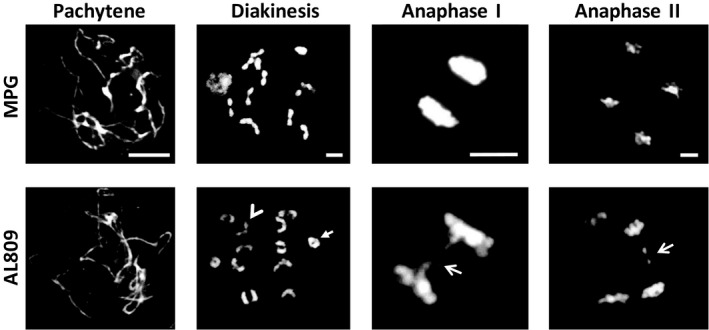
DAPI staining of chromosome spreads in various meiotic stages in wild type (MPG) and the *recq4* mutant (AL809). Arrowhead and arrow in the bottom diakinesis image point at rod and ring bivalent, respectively. Note the presence of anaphase bridges in the recq4 mutant (arrows). The bar is 10 μm.

Further analysis of meiotic stages up to pachytene showed no significant differences between wild‐type hybrid and mutant plants in the meiotic configuration of the chromosomes. However, at stages from anaphase I, the *recq4* mutant cells displayed striking aberrant features such as chromosome bridges and fragments in 32% of the cells (*n* = 1044) against 9% in wild type (*n* = 232, *P*‐value = 4.5*10^−13^; Figure 4). These aberrations indicate defects in the DSB repair. Interestingly, the aberrant features were observed during anaphase I as well as during anaphase II, suggesting that the *recq4* mutation affects both sister‐chromatid repair and homolog repair pathways. The frequency of aberrations was higher in anaphase II (40%, *n* = 331) compared with anaphase I (28%, *n* = 713). This suggests that *RECQ4* is also critical during the sister‐chromatid repair pathway or that DSB repair via the homolog repair pathway is less frequently used compared to the sister‐chromatid repair pathway. The latter would be in contrast to yeast, where DSB repair by the homologous chromosome is preferred rather than the sister chromatid (Kim *et al*., 2010).

### *RECQ4* knockout results in increased crossovers throughout the genome and extends the genetic map length

**Figure 5:**
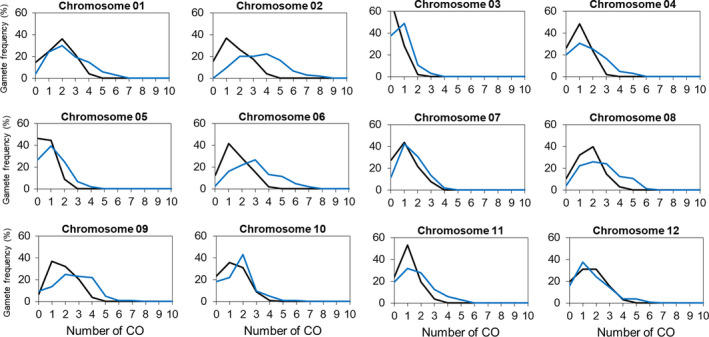
Crossover frequency distribution in wild‐type F1 and *recq4* F1 plants as measured by the transition of KASP marker genotypes from one marker position to the next, for each of the 12 chromosomes. Black lines: wild type. Blue lines: recq4 mutant.

In order to evaluate the genome‐wide effects of the *recq4* mutation on crossovers in both male and female gametophytes and to get a realistic impression of the impact in breeding terms, we measured the recombination frequency by counting crossovers in 90 genomic intervals defined by 104 polymorphic KASP markers in the F2 progeny of the wild‐type (wt) hybrid (*n* = 103) and of the *recq4* hybrid (*n* = 104). Genotype scoring tables for all F2 progeny and all good markers are shown in Table S1. Compared with the wt F1, the crossover frequency in the *recq4* mutant is shifted upwards for all chromosomes (chi‐squared test, *P* < 0.05 excluding chromosomes 10 and 12; Figure 5 and Table S2), as well as for the entire genome with on average 26.9 crossovers detected in *recq4* F1 versus 17.6 in wild type (chi‐squared test, *P* = 2.2E‐16; Table S2). On the 90 intervals between adjacent linked markers, 41 showed significant differences (chi‐squared test, df = 1, *P* < 0.05) for the crossover rates (45.6% of the intervals). In almost all cases, higher frequencies of crossovers were found in the *recq4* mutant (39/41 intervals, 95%). These intervals were mostly located on chromosome ends. For all 12 chromosomes, the numbers of detected crossovers had increased substantially and significantly in the *recq4* mutant as compared to the progeny of wt F1 plants by a factor of 1.53 (chi‐squared test with Bonferroni correction for the whole genome *P* < 2.2E‐16; Table S2).

**Figure 6:**
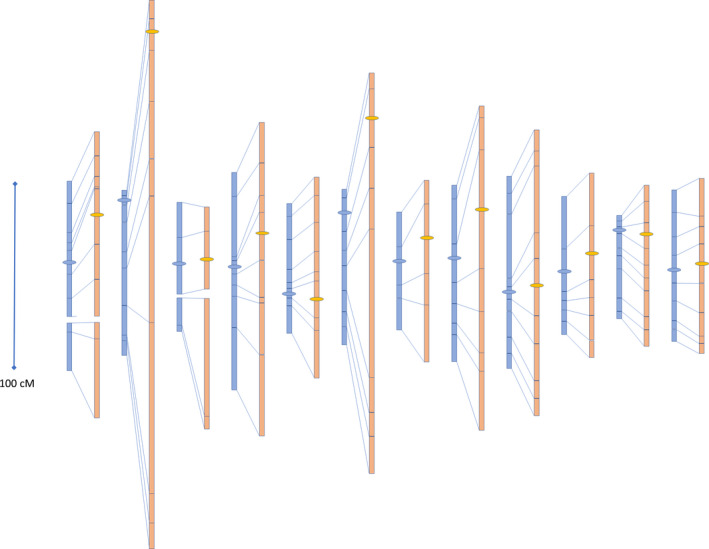
Genetic map of wild‐type (wt) F1 (left, blue) and *recq4* F1 (right, orange) of the cross between *Solanum lycopersicum* and *S. pimpinellifolium*. Lines between the wt and *recq4* maps indicate positions of the same markers. Map lengths of the individual intervals of the 12 tomato chromosomes (from left to right are chromosome 1 to 12) are shown. Horizontal ovals indicate the approximate location of the centromeres.

Construction of a genetic map using Mapmaker v3.0 and the KASP marker F2 genotyping data resulted in a cumulated genetic map of 14 linkage groups (2 each for chromosomes 1 and 3) of 997 cM for the wt F1 hybrid and one of 1766 cM estimated for the *recq4* mutant (Figure 6, Figure S3, and Table S3). These results clearly show that the *recq4* mutation produces an increased crossover frequency resulting in a 1.8‐fold extension of the genetic map.

## Discussion

Here we show the application of CRISPR/Cas9‐mutagenesis for mutating a gene involved in crossover formation and resulting in increased crossover frequency in an interspecific tomato hybrid. In the very recent study by Mieulet *et al*. (2018), it was shown that single mutations in the *RECQ4* gene increase crossovers about threefold in tomato, rice (*Oryza sativa*) and pea (*Pisum sativum*). In that study, a tomato intraspecific cross (cv. Microtom and cv. M82) was used, and crossovers were measured for two chromosomes (4 and 7). Their observed 2.7‐fold increase in recombination is higher than the 1.5‐fold increase observed by us for these two chromosomes. This may be due to the differences between intraspecific and interspecific hybrids, which suggests that the interspecific genetic background may have weakened the effect of the *recq4* mutation. The sequence divergence between the parents used here, tomato cv. Moneymaker and *S. pimpinellifolium* G1.1554 was previously estimated to amount to approximately 0.6% (Aflitos *et al*., 2014) or a more conservative 1.2–4.7 SNP/kB (Demirci *et al*., 2017). This is similar to the 5 SNPs/kB in the Col0/Ler‐hybrid (Zapata *et al*., 2016). The apparent prevention of extra crossovers in *recq4* or *fancm* mutants by sequence divergence was also suggested by others (Fernandes *et al*., 2018; Mieulet *et al*., 2018). Alternatively, the interspecific genetic background may restrict a too high increase in crossovers. Nevertheless, the results of this study demonstrate that the manipulations of anti‐crossover genes can be used to enhance recombination in both intra‐ and interspecific hybrids of crops.

Increasing meiotic crossovers in crops is of high interest because of the limitations that lack of crossovers imposes on introgressing valuable traits from wild relatives into cultivated crops. In tomato, wild relatives have large genetic diversity, especially within the self‐incompatible species like *S. chilense* and *S. peruvianum* (Bai and Lindhout, 2007). Introgression breeding in tomato, which allowed access to all the variation present in thousands of *Solanum* accessions and started approximately 80 years ago (Rick and Chetelat, 1995), has been an important strategy to broaden the genetic base of cultivated tomatoes (Bai and Lindhout, 2007). Introgression breeding relies on meiotic crossovers to produce a unique combination of favourable alleles of different species. This underlines the urgency of translating the increased knowledge of the regulation of recombination and crossover formation during meiosis obtained in the model species *Arabidopsis*, into crops such as tomato where interspecific hybridization plays a crucial role in introgression breeding.

In this study, we have investigated the role of tomato *RecQ4* in crossover formation in an interspecific cross of cultivated tomato with its wild relative *S. pimpinellifolium*. Knocking out the function of both alleles by CRISPR/Cas9‐mutagenesis in the F1 hybrid resulted in a 1.53‐fold increase of ring bivalents in male meiocytes and a 1.8‐fold extension of the genetic map. This increase does not match the sixfold increase in *Arabidopsis* Col0 *recq4a/recq4b* double mutants, or the almost fourfold increase in *Arabidopsis* Col0/Ler ‘hybrids’ reported earlier (Fernandes *et al*., 2018), suggesting a less important role for tomato RECQ4 in limiting (*inter*specific) crossovers, possibly correlated with an increased role for parallel crossover‐limiting pathways. On the other hand, a *fancm* mutation in an *Arabidopsis* hybrid (Fernandes *et al*., 2018), as well as a single mutation in a conserved amino acid of tomato *FANCM* (Mieulet *et al*., 2018)*,* acting in such a parallel pathway, did not affect crossover frequency. Alternatively, there may be residual RECQ4 activity arising from one or both mutant alleles. Contradicting the residual activity explanation is our observation of increased frequencies of aberrant chromosomes and chromosome segregation during meiosis in the *recq4* F1 mutants of tomato. This indicates that the *recq4* mutation compromises the resolution of inter‐chromosome links in meiosis I and II in an interspecific hybrid. In *Arabidopsis*, these aberrations do not occur in *recq4ab* double mutants but do happen in *recq4ab figl1* or *recq4ab fancm* triple mutants, but only slightly reduced fertility (Fernandes *et al*., 2018). Also, the observation of anaphase II bridges points at a role for RECQ4 in the sister‐chromatid pathway for DSB repair, which is not yet reported in plants, although RECQL4 helicases do play a role in sister‐chromatid cohesion (Mann *et al*., 2005).

In *Arabidopsis*, RECQ4 limits type II crossover events (Mercier *et al*., 2015). Combination of these mutations with mutations in the parallel anti‐crossover pathway involving *FANCM* further increased crossover frequency to 9 times the wild‐type frequency in a pure *Arabidopsis* line but had no effect in an *Arabidopsis* hybrid (Fernandes *et al*., 2018). Increasing the crossover frequency through increased expression of the class I‐specific E3 ligase HEI10 had a similar additive effect on crossovers in *recq4ab* mutants (Serra *et al*., 2018). Combination of a *fancm* or *recq4ab* mutation with a *figl1* mutation in *Arabidopsis* had similar additive effects (Fernandes *et al*., 2018). Higher or lower than normal temperatures increases crossover frequencies in *Arabidopsis*, presumably through the interfering class I pathway (Lloyd *et al*., 2018; Modliszewski *et al*., 2018). Selected small molecules may also reduce anti‐crossover protein activity, as was shown for the human Recq4 protein ortholog BLM (Nguyen *et al*., 2013). Combinations of such genetic interventions or treatments with mutations in tomato *RECQ4* hold promise for further improving crossover frequency in intra‐ and interspecific tomato crosses in the future.

## Experimental procedures

### Plant materials and transformation

Hybrid tomato seeds were obtained from a cross between *S. lycopersicum* cv. Moneymaker and *S. pimpinellifolium* G1.1554. Seeds from this cross were germinated under sterile conditions and transformed essentially as described (Huibers *et al*., 2013) using the *Agrobacterium tumefaciens* strain AGL1 containing the binary vector for CRISPR/Cas9‐mutagenesis described below. Rooted transformed shoots were genotyped and further grown under standard greenhouse conditions when selected for further study.

For mutagenesis of the tomato *RECQ4* ortholog, cotyledon explants of tomato hybrid seedlings were transformed with a binary vector expressing a human‐codon optimised version of *SpCas9* under transcription control of the *CaMV 35S* promoter (Brooks *et al*., 2014). This was combined by Golden Gate cloning with four modules encoding a synthetic‐guide RNA under control of a synthetic U6 promoter, into the level 2 binary vector as described elsewhere (Brooks *et al*., 2014). Selected synthetic‐guide RNA sequences, and the level 1 vectors into which they were cloned before assembly with Cas9 and an *NPTII*‐containing selection gene cassette in the level 2 vector are listed in Table S4. Leaf material was sampled from transformed seedlings, and genomic DNA was purified from these for PCR‐based screening of the targeted regions for mutations. *RECQ4* was amplified by PCR using primers as listed in Table S4 and analysed by agarose gel electrophoresis to detect deletions in the targeted regions. Bands representing the separate alleles were purified and sequenced by Sanger sequencing. Primers used to amplify the various targeted sequences are listed in Table S4.

### Cytology

Entire flower buds of the biallelic mutant and wt F1 plants were fixed in ethanol/acetic acid 3:1 and conserved for several weeks at 4 °C the fixative solution. Flower buds were rinsed three times for 5 min) in distilled water and then rinsed twice in citrate buffer (10 mm pH 4.5) for 5 min. Under a binocular microscope, flower buds were dissected to obtain the anthers (or meiotic cells) and prepared essentially according to (Zhong *et al*., 1996). Five to six anthers per plant were transferred in 50 μL of enzyme mix, consisting of 1% (w/v) cellulase, 1% (w/v) pectolyase and 1% (w/v) cytohelicase in citrate buffer (10 mm pH 4.5), at 37 °C in a moister chamber for 3 h in the dark. After sufficient digestion, the enzyme mix was removed and replaced by citrate buffer. Anthers were then macerated in the citrate buffer using a clean needle to obtain a cell suspension (bigger debris were removed). This was then transferred into an Eppendorf tube and conserved at 4 °C/on ice.

The cell suspension of 3 μL was transferred on a superfrost microscopy slide where 10 μL of 60% acetic acid solution was then added. Microscopy slides were then heated for 2 min at 45 °C, and after the first minute, another 10 μL of 60% acetic acid solution was added. During the heating, the cell suspension mix was stirred in a circle using a clean needle without touching the glass of the microscope slide in order to remove as much as possible of the cytoplasm. The cell suspension was then flooded by 3:1 fixative solution by initially adding drops of fixative all around the acetic acid drop. The slide was then tilted to remove the fixative, shaken to remove drops and let to dry. Completely dried slides were stained by adding 10 μL of DAPI (1 μg/mL) in PBS for 10 min and washed in PBS for two times 5 min. Slides were mounted with 10 μL of Vectashield and a 24 × 32 mm coverslip. Observations were made using a Zeiss Axioskop 2 microscope or with a Nikon instrument A1 confocal laser microscope. Photographs were taken using a Zeiss AxioCam 503 colour camera.

### Marker‐assisted analysis

For marker analysis in F2 plants, progenies of a mutant and wt F1 plants, the F1 itself, and the two parental lines were used. In total, 115 polymorphic SNPs selected from a previous publication (Víquez‐Zamora *et al*., 2014) were used in a KASP (Kompetitive Allele‐Specific PCR) genotyping assay performed at van Haeringen Laboratorium (Wageningen, The Netherlands). All polymorphic markers were scored (A/B/H, for parent A homozygous, parent B homozygous or heterozygote, respectively). Crossover in a specific interval in one parental (A or B) chromosome (going from homozygous A or B to heterozygous H, or vice‐versa, from one marker to the next) or crossovers in both parental chromosomes (going from homozygous A to homozygous B) was scored (marker scoring tables are provided in Data S1). Every genotype transition going from one marker to the next on the same chromosome was counted as a single crossover event in that individual F2 plant.

From the processed genotyping data, samples showing more than 10% missing data and markers having unexpected Mendelian segregation were excluded for linkage analysis. In total, 104 markers were applied on 103 and 104 individuals of the F2 populations deriving from the wt and the *recq4* mutant F1 hybrids, respectively (Table S1). Considering that variations for crossover rates were expected between F2 populations, linkage groups and marker ordering were defined based on the progeny obtained from the wt F1. The linkage analyses were performed using Mapmaker v3.0 software (Lander *et al*., 1987), by establishing linkage groups at a Logarithm of Odds Score (LOD) threshold of 4.0, and by ordering markers at a LOD of 3.0 with a maximum recombination frequency of 0.4. With these stringent parameters, 14 linkage groups were found, and the 12 expected chromosomes were obtained by reducing the LOD to 2.5. Following the validation of concordant genetic and physical location on the same chromosome for all markers, the Kosambi function was applied to evaluate the genetic distances in centimorgan (cM) between linked markers from each F2 population (Kosambi, 1943).

## Conflict of interest

The authors declare no conflict of interest.

## Author contributions

R.d.M., P.F. and Y.B. designed the studies. A.L. generated transgenic plants and mutagenesis constructs, performed genotyping and grew the plants. A.L., F.M‐D. and A.P. performed CASP genotyping and analysis. J.C. and P.F. performed cytology experiments and their analysis. R.d.M., P.F. and Y.B. wrote the manuscript with input from A.P.

## Supporting information


**Figure S1** Neighbour‐Joining tree of RECQ family protein sequences from *Arabidopsis*, grape (*Vitis vinifera*) and tomato.
**Figure S2** (a) *RECQ4* cDNA from the *recq4* F1 hybrid (left lanes) and wild‐type hybrid (right lanes) showing the single band for the wild‐type alleles’ cDNA (top right), the 1nt‐insertion allele of *Solanum pimpinellifolium* (top left), and the additional band for the 254 bp deletion of the cv. Moneymaker allele. (b) Sanger sequencing traces of the *S. pimpinellifolium* wild type and 1 nt‐insertion alleles
**Figure S3** Cumulative genetic map length in cM for the twelve chromosomes of wild‐type F1 (left, black) and *recq4*F1 (right, grey) of the *Solanum lycopersicum* x *S. pimpinellifolium* cross.
**Table S1** List of KASP markers used for obtaining the genetic maps and their physical position in the tomato genome version SL2.40
**Table S2** Statistical analysis of differences in crossover frequencies per chromosome performed through a Chi‐square test with the application of Bonferroni correction for the wild‐type (wt) F1/*recq4* F1 comparison.
**Table S3** Genetic distances in cM for each calculated marker interval compared for wild‐type F1 and *recq4* F1, respectively.
**Table S4** Guide RNA sequences, cloning vectors and PCR or sequencing primers.

**Data S1** Marker scoring tables for the wild‐type and recq4 mutant F2 progenies.
