# Supplementary material for: CRISPR/Cas inactivation of RECQ4 increases homeologous crossovers in an interspecific tomato hybrid
Source: Plant Biotechnol J. 2019 Sep 30;18(3):805–13. doi: 10.1111/pbi.13248 (PMC7004904; doi:10.1111/pbi.13248)
Supplement: Supplementary file 1 — Figure S1 Neighbour‐Joining tree of RECQ family protein sequences from Arabidopsis, grape (Vitis vinifera) and tomato. Figure S2 (a) RECQ4 cDNA from the recq4 F1 hybrid (left lanes) and wild‐type hybrid (right lanes) showing the single band for the wild‐type alleles’ cDNA (top right), the 1nt‐insertion allele of Solanum pimpinellifolium (top left), and the additional band for the 254 bp deletion of the cv. Moneymaker allele. (b) Sanger sequencing traces of the S. pimpinellifolium wild type and 1 nt‐insertion alleles Figure S3 Cumulative genetic map length in cM for the twelve chromosomes of wild‐type F1 (left, black) and recq4F1 (right, grey) of the Solanum lycopersicum x S. pimpinellifolium cross. Table S1 List of KASP markers used for obtaining the genetic maps and their physical position in the tomato genome version SL2.40 Table S2 Statistical analysis of differences in crossover frequencies per chromosome performed through a Chi‐square test with the application of Bonferroni correction for the wild‐type (wt) F1/recq4 F1 comparison. Table S3 Genetic distances in cM for each calculated marker interval compared for wild‐type F1 and recq4 F1, respectively. Table S4 Guide RNA sequences, cloning vectors and PCR or sequencing primers. [file PBI-18-805-s001.pdf]

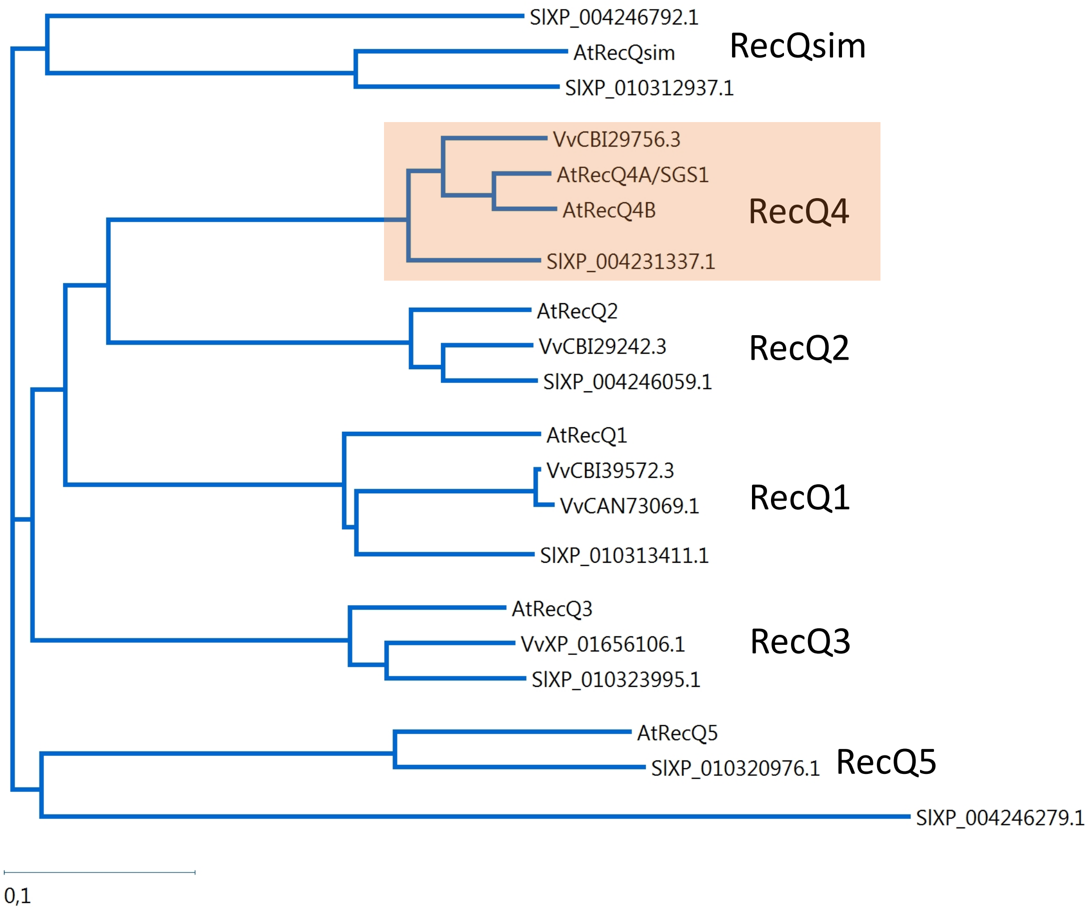

Figure S1 Neighbour-Joining tree of RECQ family protein sequences from Arabidopsis, grape (*Vitis vinifera*) and tomato. Protein sequences are named by their common name (Arabidopsis) or their Genbank accession number. XP_ numbers indicate proteins predicted from transcript models.

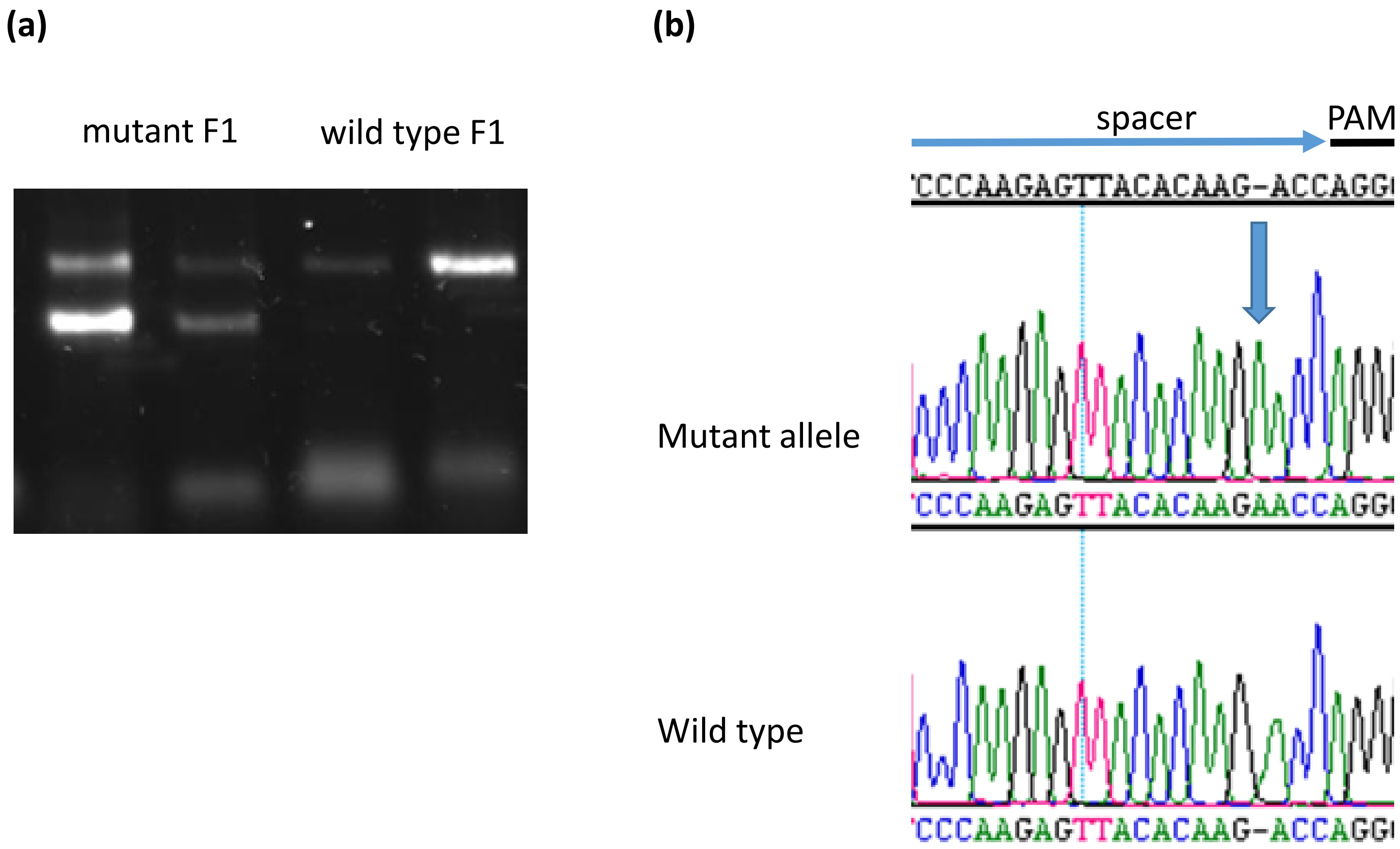

Figure S2 (a) RECQ4 cDNA from the recq4 F1 hybrid (left lanes) and wild type hybrid (right lanes) showing the single band for the wild type alleles' cDNA (top right), the 1nt-insertion allele of *Solanum pimpinellifolium* (top left), and the additional band for the 254 bp deletion of the cv. Moneymaker allele. (b) Sanger sequencing traces of the *S. pimpinellifolium* wild type and 1 nt-insertion alleles

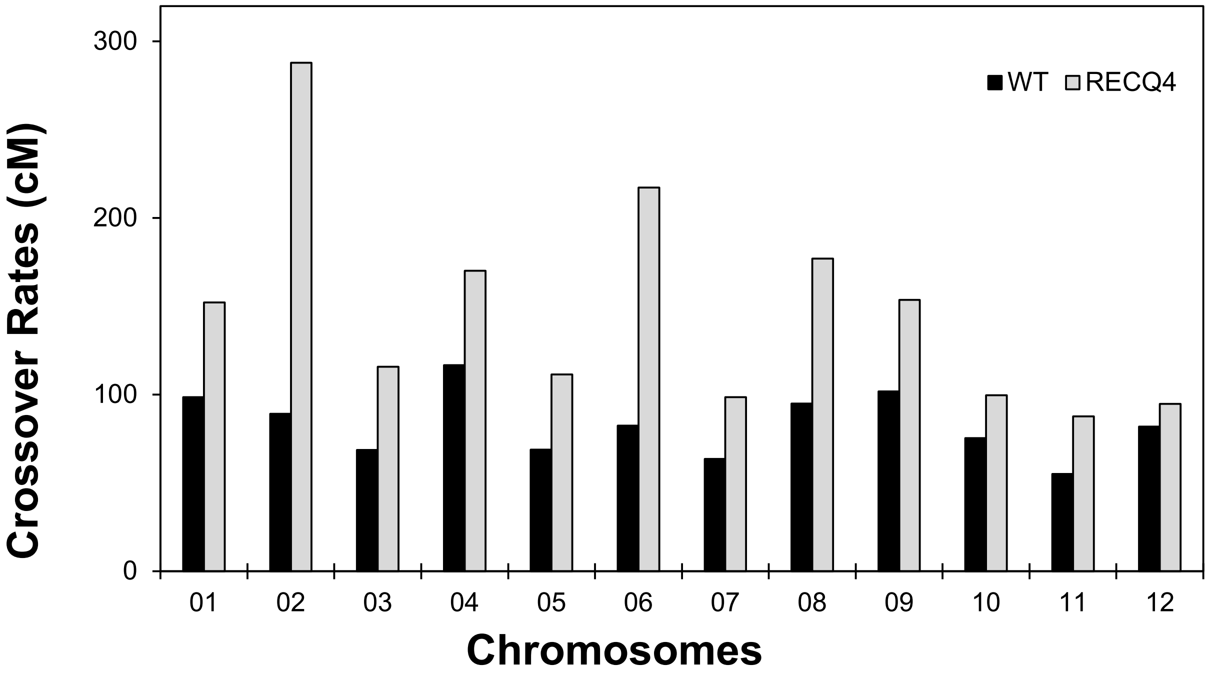

Figure S3. Cumulative genetic map length in cM for the twelve chromosomes of wild type F1 (left, black) and recq4 F1 (right, grey) of the *Solanum lycopersicum* x *S. pimpinellifolium* cross.

Table S1. List of KASP markers used for obtaining the genetic maps and their physical position in the tomato genome version SL2.4
"c" indicates the markers flanking the centromer

Marker	Chromosome	Position (Mb)	
seq-rs465	1	0.2	
sl_60356_ch0	1	1.4	
sl_60089_ch0	1	2.8	
sl_59964_ch0	1	3.5	
seq-md8273	1	3.7	c
seq-rs7523	1	69.7	c
sl_12352_ch0	1	80.0	
sl_17072_ch0	1	82.5	
seq-rs6425	1	84.5	
seq-md6450	1	85.5	
seq-rs7796	1	89.4	
sl_70010_ch0	2	2.2	
seq-rs4513	2	4.4	c
seq-md5719	2	28.5	c
seq-rs4487	2	32.3	
seq-rs5606	2	34.9	
seq-rs7406	2	36.6	
seq-md6683	2	42.5	
seq-rs8849	2	47.2	
seq-md8825	2	48.1	
seq-wv7270	2	49.5	
seq-md8481	3	0.9	
sl_101063_ch	3	2.1	c
sl_27248_ch0	3	50.3	c
seq-rs8411	3	57.7	
seq-md5556	3	61.5	
seq-rs8366	3	61.9	
seq-rs6996	4	0.2	
seq-rs4521	4	2.3	
seq-rs6567	4	4.9	
seq-rs6576	4	5.5	c
seq-rs6701	4	51.4	c
seq-rs7746	4	53.7	
sl_24605_ch0	4	55.0	
sl_11562_ch0	4	60.1	
seq-md7213	4	62.1	
seq-rs9024	5	0.3	
sl_52699_ch0	5	1.2	
sl_49002_ch0	5	3.0	
seq-rs7341	5	4.5	
seq-rs33	5	5.9	
seq-md7559	5	6.5	c
seq-rs4572	5	59.6	c
seq-rs678	5	60.6	
seq-rs6199	5	62.5	
seq-rs6370	6	0.9	
seq-md8764	6	1.6	c
seq-rs6881	6	32.1	c
seq-md3553	6	34.8	
seq-rs3977	6	36.8	
seq-rs8112	6	40.0	
seq-rs8086	6	41.5	
seq-md418	6	42.9	
seq-rs5410	6	44.9	
seq-rs8925	7	1.8	
seq-rs8899	7	3.3	c
seq-rs7703	7	57.3	c
seq-rs7764	7	59.0	
seq-rs7912	7	61.5	

Marker	Chromosome	Position (Mb)	
seq-rs400	8	0.4	
seq-rs8017	8	1.0	
seq-rs8030	8	2.9	c
seq-rs8571	8	55.8	c
seq-rs8607	8	57.7	
seq-rs8615	8	59.3	
seq-md103	8	60.0	
seq-rs11	8	62.7	
seq-rs6901	9	0.0	
seq-rs8128	9	1.3	
seq-rs6355	9	2.1	
sl_16579_ch09	9	4.6	
seq-rs6948	9	5.8	c
seq-md2744	9	55.6	c
seq-rs1913	9	60.4	
seq-rs7120	9	62.6	
seq-md435	9	64.3	
seq-rs9071	9	65.1	
seq-rs9094	9	65.8	
seq-rs7089	10	1.7	
seq-rs5755	10	21.3	c
seq-rs3687	10	61.0	c
seq-rs3691	10	61.7	
seq-rs3764	10	62.4	
seq-rs5552	10	63.7	
seq-rs5550	10	64.4	
seq-rs470	11	4.6	
seq-md8439	11	5.3	
seq-md1693	11	6.5	c
seq-rs2734	11	46.4	c
seq-rs1504	11	48.8	
seq-rs7721	11	49.8	
seq-rs7983	11	50.5	
seq-rs6903	11	51.9	
seq-rs6924	11	52.5	
seq-rs4074	11	53.2	
seq-rs7045	12	0.3	
seq-rs6474	12	1.8	
seq-rs6503	12	2.7	
seq-rs6526	12	3.8	c
seq-md851	12	32.2	c
seq-rs3433	12	47.5	
seq-rs3993	12	48.5	
seq-md3504	12	62.0	
seq-rs3519	12	62.5	

Table S2. Statistical analysis performed through a Chi-square test with application of Bonferroni correction for the wild type (wt) F1/*recq4* F1 comparison.

		Average nb. of COs			Statistics		
		**Wt F1**	***recq4* F1**	**Ratio**	**X^2**	**df**	**P-value**
**Chromosomes**	**01**	1.78	2.43	1.37	12.67	8	<0.05
	**02**	1.63	3.59	2.20	95.71	8	<0.05
	**03**	1.10	1.53	1.39	9.62	3	<0.05
	**04**	1.89	2.74	1.45	20.29	7	<0.05
	**05**	1.27	1.95	1.53	18.04	7	<0.05
	**06**	1.52	2.95	1.94	61.46	7	<0.05
	**07**	1.10	1.52	1.38	10.57	3	<0.05
	**08**	1.67	2.55	1.53	25.31	6	<0.05
	**09**	1.82	2.65	1.46	20.71	9	<0.05
	**10**	1.28	1.67	1.31	6.85	5	>0.05
	**11**	1.04	1.63	1.56	15.35	8	<0.05
	**12**	1.51	1.69	1.12	1.06	7	>0.05
**Average**		1.47	2.24	1.53			
**Whole genome**		17.61	26.90	1.53	250.37	89	2.2E-16

Table S3 Genetic distances in cM for each calculated marker interval compared for wild type F1 and for *recq4* F1, respectively
The second separated linkage groups for chromosomes 1 and 3 are indicated by grey shading.

Chr.	Mk1	Mk2	MAP with Kosambi (in cM)	
			wild type F1	*recq4* F1
	**Chr.01**		**73,3 + 25,3**	**99,6 + 52,6**
Chr.01	1	2	12	13
Chr.01	2	3	16	11
Chr.01	3	4	6	6
Chr.01	4	5	4	1
Chr.01	5	6	13	30
Chr.01	6	7	13	19
Chr.01	7	8	10	20
Chr.01	9	10	5	9
Chr.01	10	11	21	43
	**Chr.02**		**89.1**	**287.9**
Chr.02	12	13	3	1
Chr.02	13	14	5	17
Chr.02	14	15	9	28
Chr.02	15	16	16	31
Chr.02	16	17	9	20
Chr.02	17	18	20	69
Chr.02	18	19	16	93
Chr.02	19	20	3	16
Chr.02	20	21	8	14
	**Chr.03**		**49,9 + 18,8**	**44,6 + 71,2**
Chr.03	22	23	19.1	13.1
Chr.03	23	24	30.8	31.5
Chr.03	25	26	15.3	64.2
Chr.03	26	27	3.5	7.0
	**Chr.04**		**116.6**	**170.2**
Chr.04	28	29	13	22
Chr.04	29	30	33	18
Chr.04	30	31	2	9
Chr.04	31	32	7	26
Chr.04	32	33	6	20
Chr.04	33	34	6	3
Chr.04	34	35	17	28
Chr.04	35	36	34	44
	**Chr.05**		**68.9**	**111.4**
Chr.05	37	38	7	10
Chr.05	38	39	13	18
Chr.05	39	40	16	16
Chr.05	40	41	7	11
Chr.05	41	42	3	5
Chr.05	42	43	5	20
Chr.05	43	44	4	7
Chr.05	44	45	15	26
	**Chr.06**		**82.5**	**217.3**
Chr.06	46	47	5	9
Chr.06	47	48	18	32
Chr.06	48	49	10	22
Chr.06	49	50	8	22
Chr.06	50	51	13	81
Chr.06	51	52	11	19
Chr.06	52	53	7	13
Chr.06	53	54	10	20
	**Chr.07**		**63.6**	**98.5**
Chr.07	55	56	19	13
Chr.07	56	57	21	38
Chr.07	57	58	7	17
Chr.07	58	59	17	31
	**Chr.08**		**95.0**	**177.1**
Chr.08	60	61	7	7
Chr.08	61	62	25	18
Chr.08	62	63	21	65
Chr.08	63	64	11	23
Chr.08	64	65	10	23
Chr.08	65	66	6	10
Chr.08	66	67	15	32
	**Chr.09**		**101.8**	**153.6**
Chr.09	68	69	9	11
Chr.09	69	70	12	8
Chr.09	70	71	27	19
Chr.09	71	72	6	16
Chr.09	72	73	5	14
Chr.09	73	74	7	31
Chr.09	74	75	6	15
Chr.09	75	76	14	20
Chr.09	76	77	9	10
Chr.09	77	78	8	9
	**Chr.10**		**75.3**	**99.6**
Chr.10	79	80	29	29
Chr.10	80	81	24	29
Chr.10	81	82	5	10
Chr.10	82	83	5	10
Chr.10	83	84	6	13
Chr.10	84	85	7	9
	**Chr.11**		**55.0**	**87.6**
Chr.11	86	87	3	9
Chr.11	87	88	3	12
Chr.11	88	89	4	14
Chr.11	89	90	8	11
Chr.11	90	91	4	7
Chr.11	91	92	7	7
Chr.11	92	93	16	14
Chr.11	93	94	7	6
Chr.11	94	95	4	9
	**Chr.12**		**81.8**	**94.8**
Chr.12	96	97	12	21
Chr.12	97	98	13	6
Chr.12	98	99	10	9
Chr.12	99	100	15	21
Chr.12	100	101	10	16
Chr.12	101	102	11	13
Chr.12	102	103	5	4
Chr.12	103	104	7	6
	**Entire genome**		**997**	**1766**

Table S4. Guide RNA sequences, cloning vectors, and PCR and sequencing primers. Vectors and plasmids were gifts from Jonathan D. Jones, Sophien Kamoun, and Sylvestre Marillonnet. Addgene number for reference and acknowledgement of the submitter refers to www.addgene.org.

Guide	Sequence (+PAM)
RECQ4 t1	ATAAGCTTCCAAAAGCCAAC(TGG)
RECQ4 t2	AATAGCATGTTGCGTCCAGT(TGG)
RECQ4 t3	CCAAGAGTTACACAAGACCA(GGG)
RECQ4 t4	GTAGACAATTCACATGCTCG(AGG)

Vector/plasmid	Addgene number	Purpose
pICH47732:: pNOS-NPTII-tOCS)	51144	Level 1, pos. 1, *NPTII*
pICH47742::2xp35S-hCas9-tNOS	49771	Level 1, pos. 2, *Cas9*
pICH47751	48002	Level 1, pos. 3, sgRNA for t1
pICH47761	48003	Level 1, pos. 4, sgRNA for t2
pICH47772	48004	Level 1, pos. 5, sgRNA for t4
pICH47781	48005	Level 1, pos. 6, sgRNA for t3
pICH41800	48020	End linker for cloning in level 2
pAGM4723	48015	Level 2 binary plant vector

Primer	Sequence	Purpose
AL722	TTTCATGCTCATTTCCTGGAC	Forward primer for *RECQ4*
AL723	GGACAACTCTGGTGGCAAAT	Reverse primer for *RECQ4*
AL739	CCTAGTTTCAAAAGTCAACAACCA	Reverse sequencing primer
AL745	CCAACCATTTCCTTCTTGAC	Forward sequencing primer
